# Developing an Artificial Intelligence Solution to Autosegment the Edentulous Maxillary Bone for Implant Planning

**DOI:** 10.1055/s-0046-1818558

**Published:** 2026-04-10

**Authors:** Mohammad-Adel Moufti, Tharwat Alhalabieh, Kawthar Mohammad, Talal Alfailakawi, Tima Alshimmari, Sumaia Sabouni, Sebelan Danishvar

**Affiliations:** 1 College of Dental Medicine, University of Sharjah, Sharjah, United Arab Emirates; 2 Research Institute for Medical and Health Sciences, University of Sharjah, Sharjah, United Arab Emirates; 3 Department of Data Science and Artificial Intelligence, Faculty of Information Technology, Al-Ahliyya Amman University, Amman, Jordan; 4 College of Engineering, Design and Physical Sciences, Brunel University London, Uxbridge, United Kingdom

**Keywords:** artificial intelligence, cone beam computed tomography, dental implant, convolutional neural network, maxilla

## Abstract

**Objectives:**

Digital dental implant planning using panoramic radiographs and cone beam computed tomography (CBCT) imaging is labor-intensive and prone to error due to clinician fatigue, limited digital expertise, and time constraints. Artificial intelligence (AI) offers a promising solution by automating image analysis. This study aimed to develop a deep learning system for segmenting edentulous maxillary ridges to support automated implant planning.

**Materials and Methods:**

A total of 209 CBCT scans were retrieved from the University Dental Hospital Sharjah image archive (Romexis, Planmeca), of which 77 met the inclusion criteria. Manual segmentation was performed using 3D Slicer software and reviewed by a third examiner. A convolutional neural network (CNN) based on the U-Net architecture was developed using the Medical Open Network for AI (MONAI) framework. The dataset was split into training (90%) and testing (10%) sets.

**Statistical Analysis:**

Model performance was evaluated using the Dice Similarity Coefficient (DSC). Low-scoring cases (DSC <0.70) were inspected in detail to identify sources of discrepancy.

**Results:**

The 77 cases comprised 30 unilateral and 47 bilateral edentulous spaces. Most involved posterior edentulism ( *n* = 57), with fewer anterior ( *n* = 5) or combined ( *n* = 15). The model achieved a mean DSC of 76.57%. Discrepancies between manual and model segmentation mainly arose from annotators excluding narrow bone regions (<4 mm) or irregular sinus floors, and from smoothing during manual labelling. In several instances, the model provided greater anatomical precision than manual segmentation.

**Conclusions:**

The developed AI model segmented maxillary edentulous spaces with moderate-to-high accuracy. With larger, more balanced datasets and refined manual labelling protocols, this approach shows strong potential to streamline digital implant planning and enhance clinical outcomes.

## Introduction

Dental implantology has become a widely accepted solution for the replacement of missing teeth, offering both functional and aesthetic benefits. One of the most critical steps in implant treatment is presurgical planning, which involves evaluating the available bone in edentulous regions and identifying vital anatomical structures such as adjacent teeth, nerves, and sinuses. This process, commonly referred to as implant planning, directly influences implant success by guiding prosthetically driven placement and minimizing surgical complications. As Sghaireen et al (2020) highlighted, accurate assessment of bone dimensions is essential for reducing the risk of implant failure. Inaccurate identification of edentulous spans can lead to critical planning errors that impact implant placement, proximity to vital anatomical structures, and ultimately, long-term success of the prosthesis. 1 Undetected variations in bone morphology may result in implant malposition, or risk to adjacent roots, neurovascular bundles, or the sinus floor, potentially requiring revision surgery and increasing treatment cost and patient morbidity.

Traditionally, implant planning was performed using two-dimensional (2D) panoramic radiographs, which offer limited spatial information. The introduction of three-dimensional (3D) cone beam computed tomography (CBCT) significantly improved diagnostic accuracy by enabling clinicians to visualize anatomical structures across multiple planes. CBCT allows for the volumetric reconstruction of oral and maxillofacial structures with minimal distortion, thanks to the use of isotropic voxels—uniform units that preserve real-world dimensions. 2 This advancement has made CBCT the standard of care in digital implant planning.

Despite its advantages, CBCT interpretation and image segmentation remain time-consuming and technique-sensitive. Different implant planning tools exist; however, manual segmentation using these tools requires significant training and remains prone to variability and human error, relying heavily on the operator's experience, focus, and familiarity with anatomical landmarks. 3 Additionally, the cost of such specialized software and the need for ongoing clinician proficiency can limit their routine use in many dental practices.

To overcome human error, recent years have witnessed a shift towards the use of AI in dentistry in applications such as caries detection, cephalometric measurements, orthodontic planning, prosthetic design, and early oral cancer screening. 2 4 5 6 7 8 Computer vision, in particular, a field of AI, has proven especially valuable in dentistry, allowing the analysis and interpretation of medical images by simulating human visual perception through advanced algorithms. 9 One of the most effective approaches in AI for analyzing medical images is deep learning (DL), a specialized branch of machine learning (ML). Deep learning relies on artificial neural networks, particularly convolutional neural networks (CNNs), which are designed to identify and learn hierarchical patterns within complex image data. Unlike traditional ML methods, which depend on manually engineered features, CNNs automatically learns patterns from raw data, making them more adaptable to different imaging contexts, including dental applications.

Several studies have successfully applied DL to mandibular segmentation. For instance, Ibragimov and Xing (2017) used CNNs to segment organs at risk in head and neck CT images, achieving a Dice Similarity Coefficient (DSC) as high as 89.5% for the mandible. 10 Minnema et al (2019) developed a mixed-scale dense CNN for segmenting the mandible and surrounding structures in CBCT images, achieving DSC scores that were comparable to those produced by more widely used architectures like U-Net and ResNet. 11 More recently, Moufti et al. (2023) developed a U-Net-based model to segment edentulous spans in the mandible, achieving a DSC of 78%, even in cases with anatomical variation. 12 Their findings confirmed the feasibility of applying AI to support automated implant planning in partially edentulous jaws. In contrast, studies targeting the maxilla—especially its edentulous regions—are notably scarce. A recent investigation by Fontenele et al (2023) evaluated CNN-based models for segmenting the entire maxillary crest, achieving impressive accuracy (DSC: 96%). 13 However, their model focused on full-arch segmentation and did not specifically address partially edentulous spans. This distinction is critical, as partial maxillary edentulism presents unique challenges due to the irregularity of bone contours and proximity to the maxillary sinus, a structure often underrepresented or inaccurately segmented in automated systems. Unlike the mandible, the maxilla is characterized by thinner trabecular bone, lower cortical density, and a more irregular structure, making it inherently more challenging to segment accurately.

Given these challenges, there is a strong need for an AI-driven approach that can accurately segment maxillary edentulous spans while reducing reliance on manual input. Assessing the available bone, including its form, volume, and spatial relationships to critical anatomical structures, is a crucial first step in automating implant planning, as it provides the anatomical basis for further evaluation of bone quality and quantity. 14

This study proposes a deep learning approach using a CNN architecture to automate the segmentation of maxillary edentulous spans in CBCT images, marking an important step toward fully AI-assisted implant planning. It aims to expand the limited body of work on AI-assisted maxillary segmentation and evaluate the feasibility of integrating such a tool into routine clinical workflows to improve reproducibility and patient outcomes.

## Materials and Methods

### Data Collection and Selection Criteria

After obtaining ethical approval from the University of Sharjah Research Ethics Committee, a total of 209 CBCT scans were retrieved from the University Dental Hospital Sharjah using Planmeca's Romexis imaging software (maximum 120 kVp, 60 mA; exposure time 1.5–36 seconds; Field of View (FOV) 3 × 3 cm to 30 × 30 cm; voxel size 200 μm [high definition 150 μm, endodontic 75 μm]). Following initial screening, 77 scans with partial maxillary edentulism were selected based on predefined inclusion and exclusion criteria. Inclusion required edentulous ridges wider than 4 mm since this is the minimum required area to accommodate a dental implant. Scans were excluded if they showed inadequate bone height (<2 mm), as these do not represent typical indications for standard implant placement and would not be clinically appropriate targets for automated implant planning. Additionally, scans with complete maxillary edentulism were excluded because the model was designed to identify edentulous spans between existing teeth for implant planning, rather than full-arch segmentation. Furthermore, scans with unhealed extraction sockets, poor image quality, or the presence of tooth buds in the area of interest were excluded.

### Manual Segmentation Protocol

The selected CBCT images were processed and segmented using 3D Slicer software (version 5.0.3). A team of four trained examiners conducted the manual segmentation, delineating edentulous regions across axial, sagittal, and coronal planes. Segmentation of the maxillary edentulous ridges extended from the right to left maxillary tuberosities distally, with the superior boundary defined by the floor of the maxillary sinus and the inferior boundary following the crest of the alveolar ridge. Segmentation was initiated by marking edentulous areas with colored segments on multiple slices. The “Grow from Seeds” tool—based on the fast grow-cut algorithm—was applied to propagate these markings into 3D segmentations. Further refinement was done using the “Joint Smoothing” and “Scissors” tools to correct for over- or under-segmentation. The final segmentations were reviewed and exported in NIfTI format (.nii.gz).

### Quality Assurance

Prior to segmentation, all examiners received calibration training to standardize the annotation process. Each segmented case was peer-reviewed by another examiner to ensure consistency. A third reviewer conducted final approval to confirm segmentation quality.

### Model Architecture and Training

A 3D U-Net architecture was implemented, accepting volumetric CBCT patches as input and generating 3D segmentation masks of the edentulous regions as output. A CNN based on the U-Net architecture was developed ( Fig. 1 ) using the Medical Open Network for AI (MONAI) framework—a PyTorch-based platform optimized for medical image analysis. 15 The input images were processed through multiple convolutional layers to extract key spatial features, while skip connections were used to pass this critical information directly from the encoder to the decoder, allowing the network to preserve fine anatomical details and produce more accurate segmentation outputs. The model was built using the MONAI framework, an open-source deep learning platform built on PyTorch, specifically designed for medical imaging applications. It provides a comprehensive set of tools for efficient training and optimization of neural networks, making it well-suited for developing high-performance models for 3D medical image analysis. Data were used for training and testing the model in a ratio of 90:10. Of the 77 CBCT scans, 69 were used for training/validation and 8 for testing. Within the training data, 10% were randomly allocated to a validation subset for hyperparameter tuning and monitoring of model performance. The model was designed to accurately identify the 3D position and size of edentulous spaces, with a focus on preserving critical maxillary structures. Its performance was evaluated based on its ability to precisely segment edentulous regions.

**Fig. 1:**
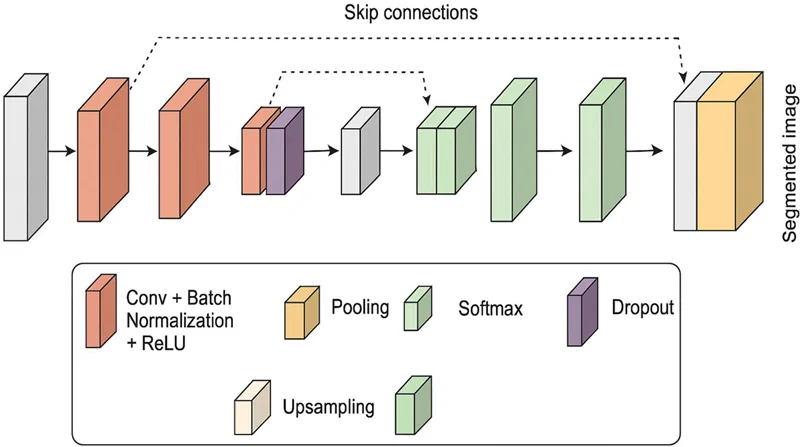
U-Net architecture used for segmenting maxillary edentulous spaces.

### Performance Evaluation

Model performance was assessed using the DSC, which quantifies the spatial overlap between model-predicted and manually segmented regions. Scores range from 0 (no overlap) to 1 (perfect overlap). Cases with DSC values below 0.70 were further examined to identify causes of discrepancy, such as anatomical variations or manual segmentation limitations.

## Results

### Dataset Summary

Based on predefined inclusion and exclusion criteria, out of 209 CBCT scans screened for eligibility, 77 cases with maxillary edentulous regions were selected for segmentation, while 132 were excluded. The final dataset included 45 males and 32 females. Edentulous areas were further categorized by laterality: Right-sided ( *n* = 18), left-sided ( *n* = 12), and bilateral ( *n* = 47). Regarding location, 5 cases involved anterior regions, 57 were posterior, and 15 involved both.

### Model Performance

The model successfully identified the edentulous spans in all tested cases, indicating reliable spatial prediction. The U-Net model was trained on 69 cases and tested on 8, yielding an overall average DSC of 76.57%. The DSC scores for the 8 test cases ranged from 0.39 to 0.85.

### Discrepancy Analysis

Thirteen cases from the entire dataset recorded DSC scores below 0.70. An in-depth review identified multiple contributing factors, as summarized in Table 1 :

**Table 1:** Causes of discrepancy between manual and automated autosegmentation

Cause of discrepancy		Number of affected cases	Notes
Model segmentation discrepancy	Sinus floor (concave extension)	2	More anatomically accurate than manual straight-line cutoff
	Tuberosity <4 mm included	6	Excluded in manual due to clinical irrelevance
	Bone over unhealed sockets	3	Low-density regions segmented by model
	Incisive canal mistakenly segmented	2	Overextension into anatomical landmark
	Metal artefact shade included	1	Caused false positive in segmentation
	Interdental bone segmented	2	Model picked up narrow bone regions not included manually
Inaccuracy of the manual segmentation	Thin bone omitted in manual segmentation	3	Missed due to visibility or tool limitations
	One area >4 mm missed manually	4	Clinical under-segmentation
	Smoothing-induced under-segmentation	7	Smoothing tool altered margins beyond acceptable thresholds

- The model more accurately followed the concave shape of the sinus floor, while manual segmentation applied a straight-line cutoff.
- Bone segments under 4 mm (e.g., in tuberosity and interdental areas) were included by the model but not manually.
- Low-density bone from unhealed sockets and artefact shadows were occasionally segmented by the model.
- Manual errors, such as missed thin bone or alterations introduced by the smoothing tool, also contributed to discrepancies.

## Discussion

Digital implant planning plays a pivotal role in presurgical assessment by identifying available bone for implant placement while avoiding adjacent vital structures. However, the manual segmentation of edentulous bone from CBCT data is time-consuming and susceptible to human error. The integration of AI, specifically through computer vision and deep learning, offers a promising approach to automating this process, potentially improving precision, efficiency, and clinical outcomes. From a clinical standpoint, the proposed AI-based segmentation model may serve as a valuable adjunct in digital implant planning. In a clinical setting, automated segmentation could substantially reduce planning time—particularly for less experienced clinicians who require guidance in identifying critical anatomical boundaries and landmarks. This can also contribute to safer surgical outcomes through consistent, high-accuracy planning and support remote treatment planning or teledentistry platforms by generating draft plans for specialist review, enhancing both speed and accessibility of implant care.

This study presents a CNN model developed using the MONAI framework to segment edentulous spans in the maxilla. This work represents the natural continuation of the AI segmentation project initiated with mandibular bone conducted by Moufti et al (2023), 12 which applied a similar U-Net CNN architecture to segment edentulous mandibular regions. Compared to the mandible, the maxilla presents additional challenges, including thinner cortical bone, proximity of the irregular sinus floor, and anatomical asymmetry, which is reflected in the slightly lower mean DSC of 76.57% versus 78% in the mandibular model. The use of DSC as the primary metric remains appropriate in this context because it directly quantifies spatial overlap between model predictions and manually labelled segmentations. 14

Manual segmentation for this study was performed using 3D Slicer, selected for its flexibility, precision, and robust support for medical imaging formats like DICOM and NIfTI. Unlike Insight Toolkit (ITK), which was used in the prior mandibular study, 3D Slicer offers more advanced tools for navigating complex anatomical structures, including the “Grow from Seeds” function for semiautomated segmentation. Its open-source design and ability to handle volumetric CBCT data also made it a practical choice for training neural networks. Despite 3D Slicer's advantages, its smoothing tool can obscure fine anatomical details, introducing variability in boundary definitions and potentially affecting the accuracy of ground truth labels. Future studies may benefit from more precise boundary refinement tools that preserve critical anatomical features without excessive smoothing.

The AI model achieved an average DSC of 76.57%. Inspection of the segmentation discrepancies between the annotators and the model revealed interesting insights. In many cases, the AI model detected anatomical structures more accurately than manual annotation. For example, in one of the highest-performing training cases, a DSC of 0.92 was achieved, indicating excellent agreement between manual and AI-assisted segmentation ( Fig. 2 ). In this instance, the model accurately captured the curved contours of the maxillary bone without overextending into adjacent soft tissues or anatomical structures, demonstrating its ability to generate clinically precise boundaries. This high accuracy reflects the model's capability to preserve fine anatomical details, even in complex regions with dense trabecular bone.

**Fig. 2:**
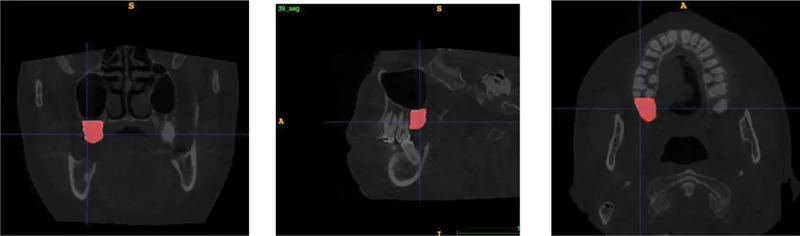
Sagittal, coronal, and transverse views of a high-performing testing case (DSC = 0.92), demonstrating excellent overlap between manual (red) and model (gray) segmentations, with accurate bone contouring and minimal artefact interference.

In contrast to the high accuracy observed in the case presented in Fig. 2 (DSC = 0.92), another case recorded one of the lowest DSC scores (0.39) due to the model incorrectly including regions affected by metal artefact shading, mistaking these visual artefacts for bone, which led to false positives and over-segmentation. Additionally, the manual segmentation overlooked a tuberosity region that should have been included based on the study's inclusion criteria, further reducing the overall overlap with the model's more anatomically comprehensive segmentation. These findings highlight the importance of high-quality, artefact-free training data and precise manual labelling protocols for improving segmentation accuracy in future studies.

Other examples of limitations in manual segmentation led to the model producing greater anatomical fidelity than manual methods, especially in complex regions such as the sinus floor or narrow ridges. For example, as shown in Fig. 3 , the model extended its segmentation into the concave sinus floor, capturing more of the natural bone contour than the manual segmentation, which followed a simplified straight-line boundary, mimicking the implantologist's work in identifying bone available up to the lowest point of the sinus. This discrepancy highlights the model's potential advantage in representing complex anatomical structures but also underscores the risk of over-segmentation if the sinus cavity itself is partially included. Similarly, Fig. 4 demonstrates a common source of mismatch associated with the use of the smoothing tool in manual segmentation. While the smoothing process helps create cleaner, more uniform boundaries, it can obscure fine anatomical details, resulting in the exclusion of thin bony ridges or cortical projections that the model correctly preserved. This trade-off between clinical practicality and anatomical precision suggests that while AI models can improve segmentation accuracy, careful consideration must be given to the effects of post-processing tools on manual training data to ensure fair performance evaluation.

**Fig. 3:**
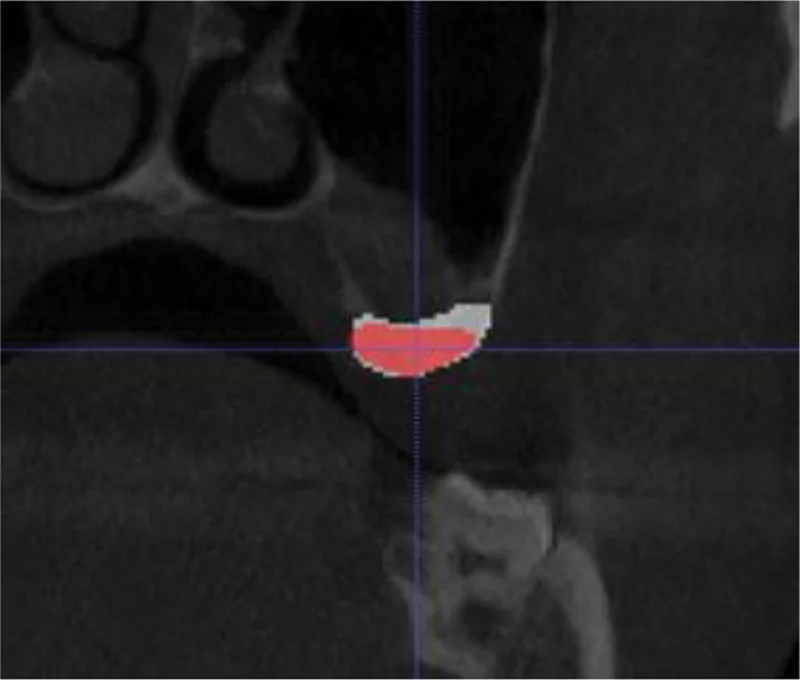
Discrepancy between manual (red) and model (gray) in sinus floor area.

**Fig. 4:**
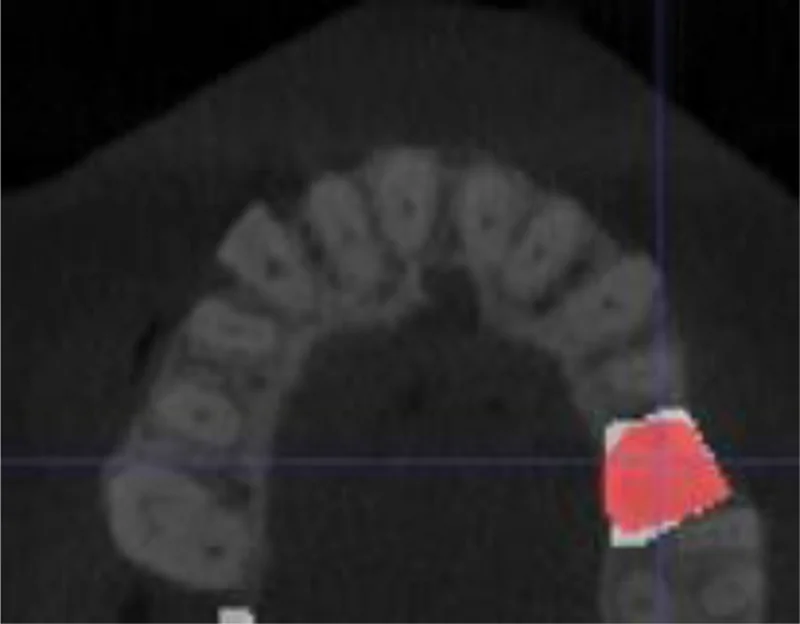
Discrepancy between manual (red) and model (gray) after the use of the smoothing tool.

The overall average DSC of 76.57% is comparable to previous work in the domain. For instance, Minnema et al (2019) 11 used a mixed-scale dense CNN to segment the mandible in CBCT images and reported mean DSC scores of 0.87 using U-Net and ResNet models. Yan et al (2018) 16 employed a symmetric CNN to segment mandibles from multi-slice computed tomography data and found that validation outcomes varied based on training–test split ratios, with optimal results at 80:13. In comparison with Fontenele et al (2023)—the only recent study to segment the entire maxillary alveolar ridge using a CNN (DSC: 96%)—the performance of the model may seem modest. 13 However, the model by Fontenele et al was trained on a commercial-grade dataset of 141 CBCTs and targeted a larger anatomical volume, making proportional discrepancies less impactful. This study, by contrast, focused on partial spans, where small differences in segmentation have a proportionally larger effect on DSC.

While the dataset included a balanced distribution of male and female patients, and segmentation sites spanned unilateral and bilateral as well as anterior/posterior locations, no clear correlation between gender or site location and model performance was observed. However, cases involving anterior regions showed a slightly higher incidence of segmentation discrepancies, likely due to underrepresentation in the training data.

Overall, our findings demonstrate that CNN-based segmentation can outperform manual efforts in complex maxillary regions. The model showed high consistency and anatomical fidelity across most cases, affirming its potential as a supportive tool in digital implant workflows.

## Study Limitations

The present study encountered several limitations related to anatomical complexity, segmentation variability, dataset size, and model evaluation. The maxilla's porous architecture and reduced bone density posed a significant challenge, particularly in areas where trabecular bone was difficult to distinguish from soft tissue. Furthermore, to standardize manual segmentation, the team used a sinus floor-aligned cutoff and excluded regions under 4 mm in width. While clinically appropriate, these criteria led to reduced overlap scores compared to the model's more anatomically comprehensive segmentation. Also, excluding fully edentulous cases may limit generalizability to completely edentulous maxillae.

Additionally, manual segmentation bias—especially due to smoothing tools used to eliminate surface irregularities—resulted in the exclusion of valid bone regions, leading to perceived discrepancies with the model output. Furthermore, the limited representation of anterior edentulous cases may have contributed to the model's occasional over-segmentation in structures such as the incisive canal.

The dataset itself was relatively small, especially in the testing phase ( *n* = 8). While similar or smaller datasets have been used in comparable work, 11 16 a larger and more diverse dataset could enhance model generalizability and reduce false positives in challenging anatomical areas.

Finally, model performance was assessed using the DSC. Complementary metrics such as precision, recall, Intersection over Union, and surface distances (95th percentile Hausdorff Distance, Average Surface Distance) were not calculated in this study; however, their inclusion in future work would provide valuable additional insights into boundary precision and clinical applicability.

## Outlook and Perspectives

To further optimize performance and clinical applicability, future studies should consider the following:

- Expand the dataset, particularly with anterior maxillary cases, to reduce anatomical bias and increase robustness.
- Enhance segmentation tools within the annotation software to provide smooth boundaries without compromising volumetric fidelity.
- Include clinician-led evaluations of model performance in real-world clinical workflows, assessing its usability and impact on treatment planning.

## Conclusion

To our knowledge, this is the first study to apply AI-assisted segmentation to partial maxillary edentulous spans using 3D CBCT data, reflecting a site-specific approach that is closer to everyday implant planning. This study successfully developed a CNN model capable of segmenting edentulous spans in the maxilla using CBCT images with a mean DSC of 76.57%, extending previous mandibular work by Moufti et al (2023) into a more complex anatomical region. 12 Despite occasional errors related to limited data and image artefacts, the model shows promising potential as a practical tool to streamline digital implant planning and reduce clinician workload in implant dentistry.

## References

[1] Sghaireen M G Srivastava K C Shrivastava D A CBCT-based three-dimensional assessment of mandibular posterior region for evaluating the possibility of bypassing the inferior alveolar nerve while placing dental implants Diagnostics (Basel) 2020 10 06 406 10.3390/diagnostics10060406 PMC7344927 32545908

[2] Lee S Oh S I Jo J Kang S Shin Y Park J W Deep learning for early dental caries detection in bitewing radiographs Sci Rep 2021 11 01 16807 10.1038/s41598-021-96368-7 PMC8376948 34413414

[3] Kernen F Kramer J Wanner L Wismeijer D Nelson K Flügge T A review of virtual planning software for guided implant surgery - data import and visualization, drill guide design and manufacturing BMC Oral Health 2020 20 01 251 10.1186/s12903-020-01208-1 PMC7488021 32912273

[4] Macr M Albis V D Albis G D The role and applications of artificial intelligence in dental implant planning Syst Rev 2024 11 08 778 10.3390/bioengineering11080778 PMC11351972 39199736

[5] Al-Asali M Alqutaibi A Y Al-Sarem M Saeed F Deep learning-based approach for 3D bone segmentation and prediction of missing tooth region for dental implant planning Sci Rep 2024 14 01 13888 10.1038/s41598-024-64609-0 PMC11180661 38880802

[6] Iacob A M Verdecchia A García-Mesa Y Spinas E Cobo T Effectiveness of automated segmentation of maxillofacial structures in cone-beam computed tomography images using artificial intelligence: a systematic review Int Orthod 2025 24 01 101081 10.1016/j.ortho.2025.101081 41066968

[7] Wang H Minnema J Batenburg K J Forouzanfar T Hu F J Wu G Multiclass CBCT image segmentation for orthodontics with deep learning J Dent Res 2021 100 09 943 949 10.1177/00220345211005338 PMC8293763 33783247

[8] Ahmed N Abbasi M S Zuberi F Artificial intelligence techniques: Analysis, application, and outcome in dentistry-A systematic review BioMed Res Int 2021 2021 9.751564E6 10.1155/2021/9751564 PMC8245240 34258283

[9] Wiley V Lucas T Computer vision and image processing: A paper review Int J Artif Intell Res 2018 2 01 29 36

[10] Ibragimov B Xing L Segmentation of organs-at-risks in head and neck CT images using convolutional neural networks Med Phys 2017 44 02 547 557 10.1002/mp.12045 PMC5383420 28205307

[11] Minnema J van Eijnatten M Hendriksen A A Segmentation of dental cone-beam CT scans affected by metal artifacts using a mixed-scale dense convolutional neural network Med Phys 2019 46 11 5027 5035 10.1002/mp.13793 PMC6900023 31463937

[12] Moufti M A Trabulsi N Ghousheh M Fattal T Ashira A Danishvar S Developing an artificial intelligence solution to autosegment the edentulous mandibular bone for implant planning Eur J Dent 2023 17 04 1330 1337 10.1055/s-0043-1764425 PMC10756774 37172946

[13] Fontenele R C Gerhardt M DN Picoli F F Convolutional neural network-based automated maxillary alveolar bone segmentation on cone-beam computed tomography images Clin Oral Implants Res 2023 34 06 565 574 10.1111/clr.14063 36906917

[14] Zou K H Warfield S K Bharatha A Statistical validation of image segmentation quality based on a spatial overlap index Acad Radiol 2004 11 02 178 189 10.1016/S1076-6332(03)00671-8 PMC1415224 14974593

[15] Cardoso M J Li W Brown R MONAI: An open-source framework for deep learning in healthcarearXiv:2211.02701 [csLG]. November 2022. Accessed April 5, 2023 at:https://arxiv.org/abs/2211.02701v1

[16] Yan M Guo J Tian W Yi Z Symmetric convolutional neural network for mandible segmentation Knowl Base Syst 2018 159 63 71

